# Editorial: ADHD and anxiety: causality sequences through a biopsychosocial model

**DOI:** 10.3389/fpsyt.2025.1627536

**Published:** 2025-05-26

**Authors:** Hongbao Cao, Shaolei Teng, Sha Liu

**Affiliations:** ^1^ School of Systems Biology, George Mason University, Fairfax, VA, United States; ^2^ Department of Biology, Howard University, Washington, DC, United States; ^3^ Department of Psychiatry, First Hospital/First Clinical Medical College of Shanxi Medical University, Taiyuan, China

**Keywords:** ADHD, anxiety, comorbidity, biopsychosocial model, Mendelian randomization

## Background and aims

ADHD and anxiety frequently co-occur, yet the directionality and mechanisms of their relationship remain debated. The biopsychosocial model posits that genetic predispositions, neurobiological processes, cognitive traits, and environmental contexts jointly shape psychopathology. Our Topic asked whether ADHD symptomatology drives anxiety (or vice versa) or if shared heritable risks underlie both? We encouraged work using longitudinal data, causal inference (e.g., Mendelian randomization), neuropsychological testing, preclinical models, and epidemiology to dissect these sequences.

## Cognitive and neuropsychological mechanisms

### Working memory and inhibitory control deficits

Kofler et al. experimentally compared competing models of working memory and inhibitory control in children with ADHD, finding that deficits in both “hot” and “cool” executive functions contribute to attentional lapses and anxiety symptoms (*Working memory and inhibitory control deficits in children with ADHD*).

### Anxiety’s impact on working memory

Marsh et al. assessed clinically evaluated children both with and without ADHD, showing that comorbid anxiety selectively impairs visuospatial working memory more than verbal spans and suggesting that anxiety amplifies ADHD’s cognitive burden (*Associations between Anxiety and Working Memory Components in Clinically Evaluated Children With and Without ADHD*).

### Developmental coordination & motor delay

Lee et al. linked developmental coordination disorder symptoms to distinct neuropsychological profiles in ADHD versus non-ADHD children, indicating that motor delays may mediate anxiety via frustrated self-efficacy (*The association between symptoms of developmental coordination disorder and neuropsychological characteristics in children with and without ADHD*).

### Preschool motor development

Cui et al. reported that early motor development delays forecast later ADHD symptom severity and anxiety traits, underscoring early neurodevelopment as a shared vulnerability window (*Association between reported ADHD symptom and motor development delay in preschool children*).

## Biological and preclinical pathways

### Fatty acids and ADHD risks

In a Mendelian randomization framework, Zhou et al. identified plasma fatty acid profiles causally linked to ADHD risk, implicating lipid-metabolism pathways that may also influence anxiety via neuroinflammation (*Plasma fatty acids and attention deficit hyperactivity disorder: a Mendelian randomization investigation*).

### Genetic & socioeconomic interplay

Deng et al. combined genetic instruments (GWAS data) with socioeconomic indicators to show that both inherited variants and environmental deprivation jointly influence risk for ADHD and anxiety, highlighting gene–environment correlation rather than pure causality (*Exploring the genetic and socioeconomic interplay between ADHD and anxiety disorders using Mendelian randomization*).

### Gene-level oncology links

Lian et al. unexpectedly uncovered gene-level connections between ADHD, anxiety disorders, and head-and-neck cancer, suggesting that pleiotropic genetic loci may underlie neurodevelopmental and somatic risks (*Gene-level connections between anxiety disorders, ADHD, and head and neck cancer*).

### Environmental enrichment in animal models

Wang et al. demonstrated that enriching the environments of neonatal rats reversed ethanol-induced attention deficits and anxiety behaviors, providing a translational model of how early interventions may reset ADHD–anxiety trajectories (*Environmental enrichment reverses prenatal ethanol exposure-induced attention-deficits in rats*).

## Conceptual and epidemiological perspectives

### Conceptual analysis of stress and anxiety

Bob and Privara reviewed neurodevelopmental disorganization processes that create vulnerability to both ADHD and anxiety, arguing for hierarchical brain-organization models to guide future treatment strategies (*ADHD, stress, and anxiety*).

### Nationwide comorbidities in Japan

Okada et al. used population-based registries to document the high prevalence of anxiety and other psychiatric comorbidities among individuals diagnosed with ADHD, underscoring that shared healthcare pathways may facilitate integrated care (*Psychiatric comorbidities of attention deficit/hyperactivity disorder in Japan: a nationwide population-based study*).

## Synthesis and future directions

Together, these studies support a multifactorial causality model in which shared genetic risks, early neurodevelopmental disruptions, executive-function deficits, and environmental contexts converge to produce overlapping ADHD and anxiety phenotypes. Mendelian randomization work (Deng et al.; Zhou et al.) bolsters the role of causal lipid and socioeconomic pathways, while preclinical enrichment models (Wang et al.) chart intervention possibilities. Cognitive investigations (Kofler et al.; Marsh et al.) clarify how anxiety amplifies core ADHD deficits, and epidemiology (Okada et al.) highlights public-health implications.

Future research should integrate longitudinal cohorts with multi-omics and digital phenotyping to map individual trajectories from infancy through adulthood. Intervention trials combining cognitive training, nutritional supplementation, and environmental enrichment merit testing. Finally, neuroimaging studies probing hierarchical brain network organization (as suggested by Bob and Privara) could illuminate shared circuit-level mechanisms.

## Conclusion

This Research Topic advances our understanding of ADHD–anxiety comorbidity by integrating genetic, neurobiological, cognitive, and environmental perspectives. By unraveling complex causality sequences, these contributions pave the way for precision interventions that address both disorders simultaneously, ultimately improving outcomes for individuals across their lifespan.

